# Cystic neuroendocrine tumor in the pancreas detected by endoscopic ultrasound and fine-needle aspiration: a case report

**DOI:** 10.1186/1756-0500-7-510

**Published:** 2014-08-09

**Authors:** Henrik Thorlacius, Evangelos Kalaitzakis, Gabriele Wurm Johansson, Otto Ljungberg, Olle Ekberg, Ervin Toth

**Affiliations:** 1 Department of Clinical Sciences, Malmö, Section of Surgery, Skåne University Hospital, Lund University, Malmö, 20502, Sweden; 2 Gastroenterology, Lund University, Malmö, Sweden; 3 Pathology, Lund University, Malmö, Sweden; 4 Radiology, Skåne University Hospital, Lund University, 20502 Malmö, Sweden

## Abstract

**Background:**

Pancreatic neuroendocrine tumors are typically solid neoplasms but in very rare cases present as cystic lesions. The diagnosis of cystic tumors in the pancreas is extremely difficult and the use of endoscopic ultrasound and fine-needle aspiration might be helpful in the work-up of patients with cystic neuroendocrine tumors in the pancreas.

**Case presentation:**

A 78-year-old Caucasian man was admitted with a history of epigastric pain. Laboratory tests were normal. The patient underwent transabdominal ultrasound, computed tomography and magnetic resonance cholangiopancreatography demonstrating an unclear cystic mass in the head of the pancreas. The patient was referred for endoscopic ultrasound. Endoscopic ultrasound showed a hypoechoic lesion (42 × 47 mm) in the head of the pancreas with regular borders and large cystic components. The main pancreatic duct was normal without any connection to the cystic process. The lesion underwent fine-needle aspiration (22 Gauge). Cytological examination demonstrated cohesive groups of plasmacytoid cells staining positively for synaptophysin and chromogranin A, which is suggestive of a neuroendocrine tumor.

**Conclusions:**

Differential diagnosis of cystic lesions in the pancreas is very difficult with conventional radiology, such as computed tomography and magnetic resonance imaging. This unusual case with a pancreatic cystic neuroendocrine tumor highlights the clinical importance of endoscopic ultrasound in the work-up of patients with unclear lesions in the pancreas.

## Background

Pancreatic cystic lesions pose a major challenge to clinicians. Due to increased use of cross-sectional abdominal imaging, cystic lesions in the pancreas have become a common incidental finding [1]. More than 60% of pancreatic cystic lesions are neoplastic [2]. In fact, neoplastic cysts constitute about 15% of all neoplasms in the pancreas [3]. Neoplastic cysts in the pancreas include intraductal papillary mucinous neoplasm, mucinous cystic neoplasm, solid-pseudopapillary neoplasm, serous cystic neoplasm, ductal adenocarcinoma with cystic degeneration and cystic neuroendocrine tumor [4]. Differential diagnosis is difficult but with the help of endoscopic ultrasound and fine-needle aspiration improved visualization and tissue sampling are possible. Herein, we describe a case with a cystic neuroendocrine tumor in which endoscopic ultrasound facilitated final diagnosis.

## Case presentation

**Figure 1:**
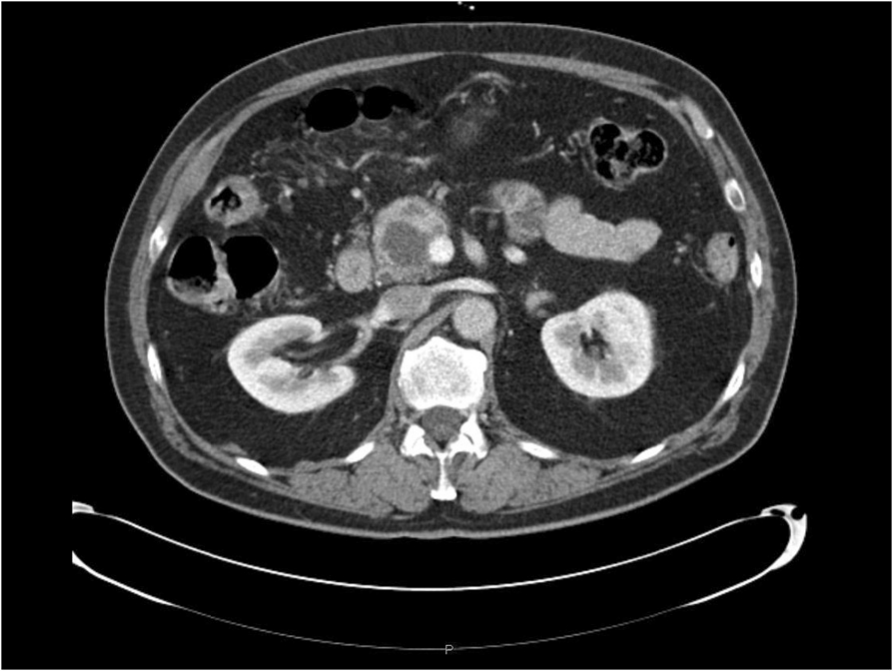
Abdominal computer tomography showing the lesion in the head of the pancreas.

**Figure 2:**
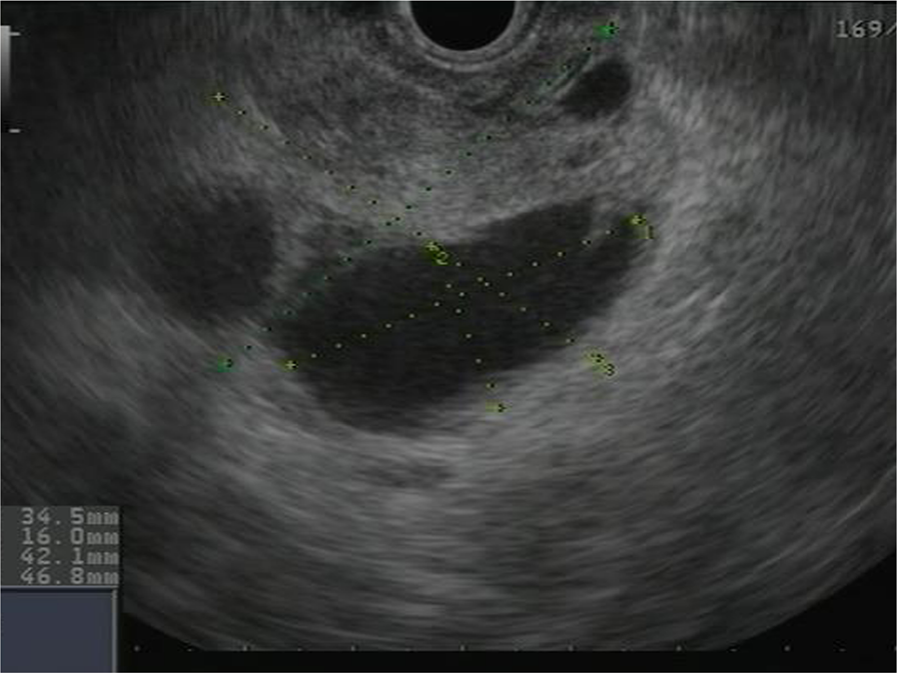
Endoscopic ultrasound image revealing a cystic lesion with regular well-demarked borders in the head of pancreas.

**Figure 3:**
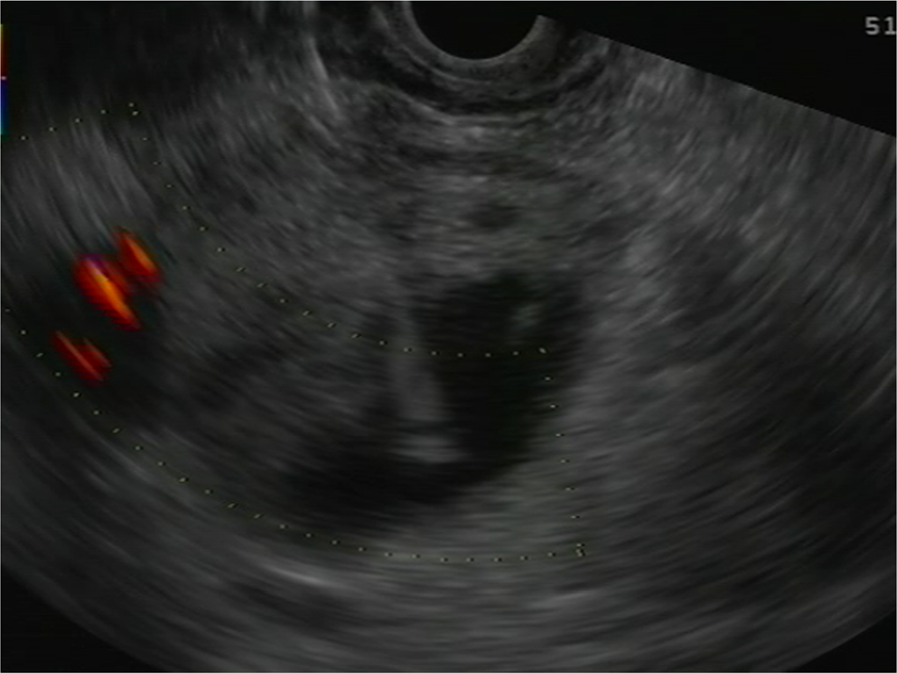
**Endoscopic ultrasound image showing the fine-needle aspiration (22 Gauge) of the cystic tumor.** The tip of the needle is in the cystic component of the lesion.

**Figure 4:**
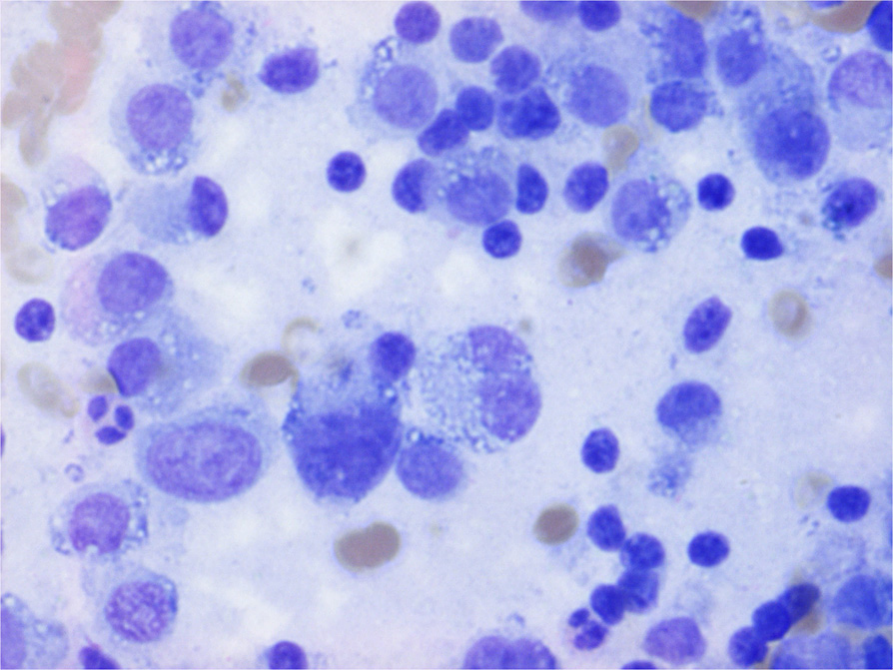
The lesion underwent fine-needle aspiration and cytological examination revealed cohesive groups of plasmacytoid cells.

**Figure 5:**
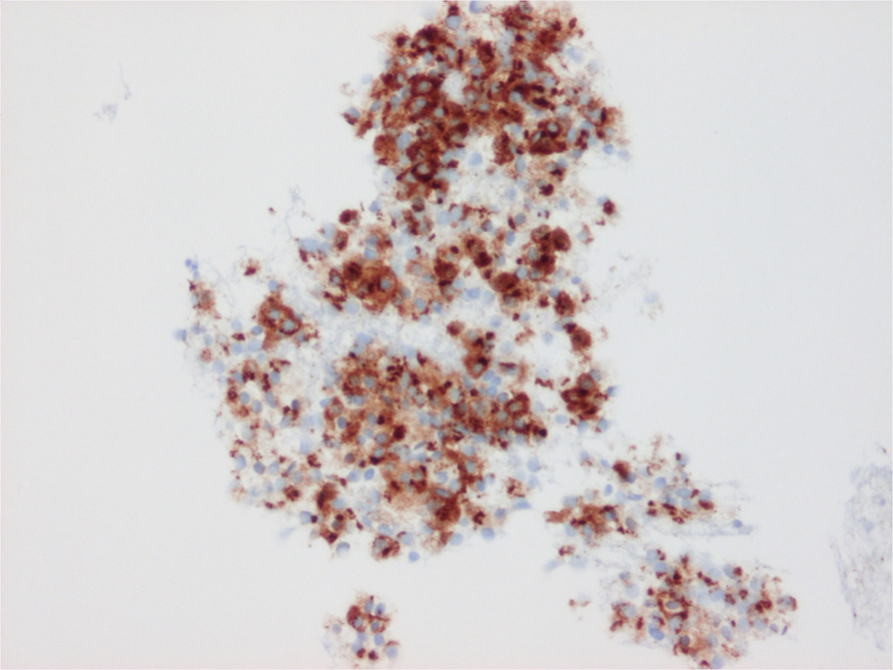
The lesion underwent fine-needle aspiration and revealed positive immunocytological expression of chromogranin A in the plasmacytoid cells.

A 78-year-old Caucasian man presented with epigastric pain. He was otherwise healthy except mild hypertension and asthma and laboratory tests were normal. Transabdominal ultrasound revealed a suspicious lesion in the head of the pancreas measuring 4 cm. A subsequent abdominal contrast-enhanced computed tomography depicted an unclear lesion as a mass (4 × 5 cm) with heterogeneous attenuation containing both solid and cystic components without signs of bile duct or pancreatic duct dilatation although engagement of the superior mesenteric vein was suspected (Figure 1). A magnetic resonance cholangiopancreatography did not add further diagnostic information and the patient was referred for endoscopic ultrasound. Endoscopic ultrasound demonstrated a hypoechoic lesion measuring 42 × 47 mm in the head of the pancreas with regular borders and large cystic components (Figure 2). The diameter of the pancreatic duct was normal and there was no morphological connection between the process and the pancreatic duct. Both the cystic and the solid components of the process underwent fine-needle aspiration (22 Gauge) (Figure 3). The fluid from the cystic component had low viscosity and contained normal levels of carcinoembryonic antigen (<1 μg/l) and amylase (2.5 μKat/l). Cytological examination of the aspirate from the solid component showed cohesive groups of plasmacytoid cells (Figure 4), staining positively for synaptophysin and chromogranin A (Figure 5), which are highly specific markers for neuroendocrine tumors [5]. Ki-67 index was less 2% making this a G1-type tumor but the engagement of the superior mesenteric vein made surgery non-optional and the patient received palliative chemotherapy.

## Discussion

Neuroendocrine tumors account for only 1-2% of all pancreatic neoplasms and less than 10% of all neuroendocrine tumors are cystic, which makes cystic neuroendocrine lesions very rare [6]. The mean age at diagnosis is 53 years and there is no evident gender predilection [7,8]. Most cystic neuroendocrine tumors in the pancreas are non-functional and the diagnosis is usually made incidentally or secondary to mass-dependent symptoms, such as abdominal pain, nausea or weight-loss, as was the case in our patient. Except for neuroendocrine microadenomas, all neuroendocrine tumors are considered to have malignant potential and should be considered for surgical resection [9,10]. However, identification of cystic neuroendocrine tumors in the pancreas with conventional axial imaging, such as computed tomography and magnetic resonance imaging, is extremely difficult [11]. Differential diagnosis includes simple cysts, other cystic neoplasms, pseudocysts and adenocarcinomas with cystic degeneration.

Endoscopic ultrasound coupled with fine-needle aspiration has enabled not only the detailed examination of pancreatic cystic lesions but also cytological, biochemical and immunocytological analysis [12]. A previous report by Kongkam *et al.*[6] could not identify any unique endoscopic ultrasound finding in cystic neuorendocrine tumors although others have suggested that cystic neuorendocrine tumors exhibit more frequently a thick wall compared to mucinous cysts [12]. Anyhow, the most important advantage with endoscopic ultrasound is the possibility to obtain tissue and fluid samples from the cysts. Several studies have shown that endoscopic ultrasound-guided immunocytology with staining for neuroendocrine markers, such as synaptophysin and chromogranin A, is an accurate method to establish the diagnosis of cystic neuroendocrine tumors [6,11], which was key to diagnosis also in the present case. In our patient, the cyst fluid levels of carcinoembryonic antigen were normal. Elevated levels of carcinoembryonic antigen in cysts are a useful marker of mucinous cysts [13,14] and it is therefore interesting to note that numerous previous studies have reported that cyst fluid levels of carcinoembryonic antigen are usually low in cystic neuroendocrine tumors [15,16].

## Conclusion

This unusual case with a cystic neuroendocrine tumor in the pancreas underlines the clinical impact of endoscopic ultrasound in the work-up of patients with unclear lesions in the pancreas.

## Consent

Written informed consent was obtained from the patient for publication of this Case Report and any accompanying images. A copy of the written consent is available for review by the Editor-in-Chief of this journal.

## Competing interests

The authors declare that they have no competing interests.

## Authors’ contributions

HT and GWJ performed the endoscopic ultrasound. HT wrote the first draft. OJ performed the histology and immunostaining. OE performed the computed tomography and magnetic resonance cholangiopancreatography. HT wrote the first draft. All authors contributed to the final manuscript. All authors read and approved the final manuscript.
